# Selective eating: searching for clarity in complexity

**DOI:** 10.1186/2050-2974-2-S1-O53

**Published:** 2014-11-24

**Authors:** Carol Smith, Neil McLean, Julie McCormack

**Affiliations:** 1 Eating Disorders Program, Specialised Child and Adolescent Mental Health Service, Department of Health in Western Australia, Perth, Australia; 2 University of Western Australia, Perth, Australia

## 

Selective Eating (SE) refers to those children who significantly restrict their eating, but demonstrate no apparent concerns about weight or shape. Typically children with SE eat a very limited range of foods, usually over a number of years. In severe cases of SE there is anecdotal evidence that the child's growth is affected, but more commonly parents report that the child's eating patterns result in marked family distress and conflict as well as impairment in social functioning. This category of eating disorders is diagnostically unclear, with no formal criteria, and a paucity of empirical research. This research set out to understand more clearly the nature of SE starting with investigation of the level of concern about the problem and associated demographic factors. An objective measure of SE was developed based on analysis of food diaries, which was crucial in both ongoing research and assessing this ill-defined problem. Finally psychological aspects of the problem were investigated by comparing children with SE with a control group. Parents of SE's were more stressed and also more involved with their child's eating. The children themselves often had a history of developmental issues such as gagging or sensory aversion, but also had an anxious temperament and behavioural difficulties when younger, and features of anxiety and particularly OCD when older. These results highlight the complexity in assessing and treating this phenomenon.

This abstract was presented in the **Service Initiatives: Child and Adolescent** stream of the 2014 ANZAED Conference.

